# ﻿Hidden in the jungle of Vietnam: a new species of *Quasipaa* (Amphibia, Anura, Dicroglossidae) from Ngoc Linh Mountain

**DOI:** 10.3897/zookeys.1124.89282

**Published:** 2022-10-10

**Authors:** Cuong The Pham, Chung Van Hoang, Tien Quang Phan, Truong Quang Nguyen, Thomas Ziegler

**Affiliations:** 1 Institute of Ecology and Biological Resources, Vietnam Academy of Science and Technology, 18 Hoang Quoc Viet Road, 10072 Hanoi, Vietnam Institute of Ecology and Biological Resources, Vietnam Academy of Science and Technology Hanoi Vietnam; 2 Graduate University of Science and Technology, Vietnam Academy of Science and Technology, 18 Hoang Quoc Viet Road, 10072 Hanoi, Vietnam Graduate University of Science and Technology Hanoi Vietnam; 3 Forest Resources and Environment Center, 300 Ngoc Hoi Road, Thanh Tri, 10000 Hanoi, Vietnam Forest Resources and Environment Center Hanoi Vietnam; 4 AG Zoologischer Garten Köln, Riehler Strasse 173, D-50735 Cologne, Germany AG Zoologischer Garten Köln Cologne Germany; 5 Institute of Zoology, University of Cologne, Zülpicher Strasse 47b, D-50674 Cologne, Germany University of Cologne Cologne Germany

**Keywords:** Kon Tum Province, molecular phylogeny, *Quasipaataoi* sp. nov., taxonomy

## Abstract

A new species of *Quasipaa* is described from Ngoc Linh Mountain of the Kon Tum Massif in central Vietnam. The new species is morphologically distinguishable from its congeners on the basis of a combination of the following diagnostic characters: SVL 79.6–84.3 mm in males and 64.6–69.9 mm in females; head broader than long; vomerine teeth present; external vocal sacs absent; tympanum slightly visible; dorsum with lines of thick ridges and small round tubercles; flanks covered by oval and round tubercles; supratympanic fold present; dorsolateral fold absent; ventrolateral sides, ventral surface of arms, and all fingers with spines in males; the absence of spines on chest and belly in males; toes fully webbed to distal portion of terminal phalanx; in life, dorsum dark brown, chest and belly immaculate white. Phylogenetic analyses found that the genetic divergence of the new species and its congeners ranged from 4.2–5.1% (compared with *Quasipaaboulengeri*) to 7.6–8.1% (compared with *Q.shini*) in the 16S gene.

## ﻿Introduction

The genus *Quasipaa* Dubois, 1992 is known from China through the Indochina region and south to Thailand. The genus currently contains 12 recognized species, including *Quasipaaacanthophora* Dubois & Ohler, 2009, *Q.boulengeri* (Günther, 1889), *Q.courtoisi* (Angel, 1922), *Q.delacouri* (Angel, 1928), *Q.exilispinosa* (Liu & Hu, 1975), *Q.fasciculispina* (Inger, 1970), *Q.jiulongensis* (Huang & Liu, 1985), *Q.robertingeri* (Wu & Zhao, 1995), *Q.shini* (Ahl, 1930), *Q.spinosa* (David, 1875), *Q.verrucospinosa* (Bourret, 1937), and *Q.yei* (Chen, Qu & Jiang, 2002) (Frost 2022). Recent phylogenetic studies have shown that there are still several unnamed distinct lineages in the genus, indicating that its species richness remains underestimated (Che et al. 2009, 2010; Yan et al. 2021).

During our recent fieldwork in the Central Highlands of Vietnam, specimens of *Quasipaa* were collected in the evergreen forests of Ngoc Linh Mountain, Kon Tum Province. These specimens were identified as members of the “*Quasipaa* sensu stricto” species group (Group II-2) (Che et al. 2010) and *Quasipaa* sp. 1 (Yan et al. 2021) based on molecular data. Closer morphological examination showed that the population from Ngoc Linh Mountain in the Central Highlands of Vietnam could be clearly distinguished from other *Quasipaa* species by a combination of morphological features. Also, in phylogenetic analyses, this taxon was clearly separated from its congeners. Therefore, we describe here the unnamed taxon from the Central Highlands of Vietnam, based on our integrative taxonomical analyses, as a new species.

## ﻿Materials and methods

### ﻿Sampling

A field survey was conducted in March 2019 in Ngoc Linh Nature Reserve, Dak Giei District, Kon Tum Province. Frogs were collected between 19:00 and 23:00. After taking photographs of living specimens, they were anaesthetized and euthanized in a closed vessel with a piece of cotton wool containing ethyl acetate (Simmons 2002), fixed in 80% ethanol for 5 h, and later transferred to 70% ethanol for permanent storage. Tissue samples were preserved separately in 70% ethanol prior to fixation. Voucher specimens referred to in this paper were deposited in the collections of the Institute of Ecology and Biological Resources (**IEBR**), Hanoi, Vietnam.

### ﻿Molecular data and phylogenetic analyses

In this study, 15 samples of five species of *Quasipaa* were used for molecular analysis (Table 1). Tissue samples were extracted using PureLink™ RNA Micro Scale Kit (Thermo Fisher Scientific company), following the manufacturer’s instructions. DNA was amplified using PCR Applied Biosystems. PCR volume consisted of 25 μl, including 12 μl of Mastermix, 6 μl of water, 1 μl of each primer at concentration of 10 pmol/μl, and 5 μl of DNA. A fragment of the mitochondrial gene (16S) with ~570 base pairs length was amplified using the primer pair LR-N-13398 (5´-CGCCTGTTTACCAAAAACAT-3´; forward) and LR-J 12887 (5´-CCGGTCTGAACTCAGATCACGT-3´; reverse) (Simon et al. 1994). PCR conditions: 94 °C for 5 min of initial denaturation; with 35 cycles of denaturation at 94 °C for 30 s, annealing at 56 °C for 30 s, and extension at 72 °C for 45 s; and the final extension at 72 °C for 7 min. PCR products were sent to Apical Scientific company for sequencing (https://apicalscientific.com).

**Table 1.** GenBank accession numbers and associated samples that used in this study.

	Species	Location	Genbank No.	References
1	*Quasipaataoi* sp. nov.	Kon Tum, Vietnam	OP326684	This study
2	*Quasipaataoi* sp. nov.	Kon Tum, Vietnam	OP326685	This study
3	*Quasipaataoi* sp. nov.	Kon Tum, Vietnam	EU979804	Che et al. (2009)
4	*Quasipaataoi* sp. nov.	Xekong, Laos	EU979803	Che et al. (2009)
5	*Q.verrucospinosa*	Vinh Phuc, Vietnam	EU979813	Che et al. (2009)
6	*Q.verrucospinosa*	Tuyen Quang, Vietnam	OP326686	This study
7	*Q.verrucospinosa*	Tuyen Quang, Vietnam	OP326687	This study
8	*Q.verrucospinosa*	Tuyen Quang, Vietnam	OP326688	This study
9	*Q.spinosa*	Yunnan, China	DQ118480	Che et al. (2009)
10	*Q.robertingeri*	Sichuan, China	EU979814	Che et al. (2009)
11	*Q.robertingeri*	Sichuan, China	DQ118478	Che et al. (2009)
12	*Q.boulengeri*	Cao Bang, Vietnam	OP326689	This study
13	*Q.boulengeri*	Cao Bang, Vietnam	OP326690	This study
14	*Q.boulengeri*	Cao Bang, Vietnam	OP326691	This study
15	*Q.boulengeri*	Cao Bang, Vietnam	OP326692	This study
16	*Q.boulengeri*	Cao Bang, Vietnam	OP326693	This study
17	*Q.exilispinosa*	Fujian, China	DQ118484	Che et al. (2009)
18	*Q.jiulongensis*	Fujian, China	KF199149	Zhang et al. (2018)
19	*Q.acanthophora*	Lang Son, Vietnam	OP326694	This study
20	*Q.acanthophora*	Lang Son, Vietnam	OP326695	This study
21	*Q.yei*	Henan, China	DQ118488	Che et al. (2009)
22	*Q.shini*	Guangxi, China	DQ118487	Che et al. (2009)
23	*Quasipaa* sp.	Xekong, Laos	EU979812	Che et al. (2009)
24	*Q.delacouri*	Tuyen Quang, Vietnam	OP326696	This study
25	*Q.delacouri*	Tuyen Quang, Vietnam	OP326697	This study
26	*Q.delacouri*	Tuyen Quang, Vietnam	OP326698	This study
	Outgroup			
	*Fejervaryalimnocharis*	Vinh Phuc, Vietnam	EU979847	Che et al. (2009)

In addition, we used 11 available sequences of 16S rRNA of nine species of the genus *Quasipaa* in GenBank for phylogenetic analyses (Che et al. 2009; Zhang et al. 2018). A sequence of *Fejervaryalimnocharis* was included in the analysis as the outgroup (Che et al. 2009). For locality information and accession numbers for all sequences used in this study, see Table 1.

Phylogenetic trees were constructed by using maximum likelihood (ML) and Bayesian inference (BI). Chromas Pro software (Technelysium Pty Ltd., Tewantin, Australia) was used to edit the sequences, which were aligned using the ClustalW (Thompson et al. 1997) option in MEGA X (Kumar et al. 2018) with default parameters and subsequently optimized manually in BioEdit v. 7.0.5.2 (Hall 1999). We then checked the initial alignments by eye and adjusted slightly. Prior to ML and Bayesian phylogenetic analyses, we chose the optimum substitution models for entire sequences using Kakusan 4 (Tanabe 2011) based on the Akaike information criterion (AIC). The BI was performed in MrBayes v. 3.2 (Ronquist et al. 2012). The BI summarized two independent runs of four Markov Chains for 10 million generations. A tree was sampled every 100 generations and a consensus topology was calculated for 70 000 trees after discarding the first 30 001 trees (burn in = 3 000 000) (Nguyen et al. 2017). We checked parameter estimates and convergence using Tracer v. 1.7.1 (Rambaut et al. 2018). The strength of nodal support in the ML tree was analyzed using non-parametric bootstrapping (MLBS) with 1000 replicates. We regarded tree nodes in the ML tree with bootstrap values of 75% or greater as sufficiently resolved (Hillis and Bull 1993; Huelsenbeck and Hillis 1993), and nodes with a BPP of 95% or greater as significant in the BI analysis (Leaché and Reeder 2002).

### ﻿Morphological characters

Measurements were taken with digital calipers to the nearest 0.1 mm. The following abbreviations were used:

**SVL** snout–vent length;

**HL** head length (measured as a parallel line with the vertebral column from posterior margin of mandible to tip of snout);

**HW** maximum head width (at rictus);

**RL** rostral length (from anterior corner of orbit to tip of snout);

**NS** distance from nostril to tip of snout;

**EN** distance from anterior corner of orbit to nostril;

**IND** internarial distance;

**IOD** interorbital distance;

**ED** eye diameter;

**UEW** maximum width of upper eyelid;

**DAE** distance between anterior margins of orbits;

**DPE** distance between posterior margins of orbits;

**MN** posterior margin of mandible to nostril;

**MFE** posterior margin of mandible to anterior margin of orbit;

**MBE** posterior margin of mandible to posterior margin of eye;

**TD** tympanum diameter;

**TYE** distance from anterior margin of tympanum to posterior corner of orbit;

**UAL** upper arm length (from axilla to elbow);

**FAL** forearm length (from elbow to tip of third finger);

**FL1–4** finger length I–IV (from inner to outer);

**NPL** nuptial pad length - finger I;

**FeL** femur length (from vent to knee);

**TbL** tibia length (from knee to tarsus);

**TbW** maximum tibia width;

**FoL** foot length (from tarsus to tip of fourth toe);

**TL** 1–5 toe length I–V;

**IMT** inner metatarsal tubercle length.

For webbing formula, we followed Glaw and Vences (2007). Sex was determined by gonadal inspection.

Morphological data were obtained by comparison of the new species with specimens of other members of the genus *Quasipaa* (see Appendix 1) and from literature (e.g., Angel 1928; Bourret 1937, 1942; Liu 1950; Inger 1970; Liu and Hu 1975; Huang and Liu 1985; Wu and Zhao 1995; Inger et al. 1999; Chen et al. 2002; Ohler and Dubois 2006; Dubois and Ohler 2009; Fei et al. 2009, 2012).

### ﻿Principal component analysis (PCA)

Measurements were used to compare the morphometric difference between the new species from Kon Tum Province (three males and three females) vs *Quasipaaboulengeri* from Cao Bang Province (six males and five females). All statistical analyses were performed using PAST v. 2.17b software (Hammer et al. 2001).

## ﻿Results

### ﻿Phylogenetic analyses

The combined matrix contained 495 aligned characters. Of those, 416 sites were conserved, and 78 sites were variable, of which 62 were found to be potentially parsimony informative. The estimated Transition/Transversion bias (R) is 3.86. Substitution pattern and rates were estimated under the Tamura (1992) model. The nucleotide frequencies are A = 26.91%, T/U = 26.91%, C = 23.09%, and G = 23.09%. In terms of pairwise genetic distance, interspecific uncorrected *p*-distance of the *Quasipaa* species ranged from 1.4% (between *Quasipaa* sp. from Laos and *Q.delacouri*), 1.6–1.9% (between *Q.boulengeri* and *Q.robertingeri*) to 7.6–8.1% (between *Q.shini* and the new form) (Table 2). The genetic divergence of the new form and its congeners ranged from 4.2–5.1% (*Q.boulengeri*) to 7.6–8.1% (*Q.shini*), which was greater than genetic distances between *Q.boulengeri* and *Q.robertingeri* (1.6–1.9%); between *Q.boulengeri* and *Q.jiulongensis* (3.8–4.0%); and between *Q.boulengeri* and *Q.delacouri* (4.0–4.5%) (Table 2).

**Table 2.** Uncorrected *p*-distance matrix showing percentage pairwise genetic divergences (%) for the 16SrRNA gene between members of the genus *Quasipaa*.

	Species	1	2	3	4	5	6	7	8	9	10	11	12
1	*Quasipaataoi* sp. nov.	**0.0**–**0.8**											
2	*Q.verrucospinosa*	6.4–7.1	**0.0**–**0.2**										
3	*Q.spinosa*	6.7–7.1	3.1–3.3	**0.0**									
4	*Q.robertingeri*	5.3–5.8	5.3–5.5	4.4	**0.0**								
5	*Q.boulengeri*	4.2–5.1	5.1–5.8	4.6–4.9	1.6–1.9	**0.0**–**0.4**							
6	*Q.exilispinosa*	6.7–6.9	6.5–6.7	6.7	6.2	4.9–5.1	**0.0**						
7	*Q.jiulongensis*	4.9–5.5	5.5–5.8	5.8	4.7	3.8–4.0	3.3	**0.0**					
8	*Q.acanthophora*	6.2–6.9	7.1–7.6	7.8–8.0	7.1–7.4	5.8–6.2	2.7–2.9	3.3–3.5	**0.0**–**0.2**				
9	*Q.yei*	5.8–6.4	6.0–6.2	6.4	5.1	4.7–4.9	5.8	5.1	5.3–5.5	**0.0**			
10	*Q.shini*	7.6–8.1	7.4–7.6	7.8	6.4	6.2–6.7	7.2	6.0	7.0–7.2	5.6	**0.0**		
11	*Quasipaa* sp.	4.9–5.6	6.0–6.2	6.2	4.7	3.6–4.0	5.1	3.4	4.2–4.4	4.5	5.6	**0.0**	
12	*Q.delacouri*	4.9–5.8	5.8–6.0	6.7	5.1	4.0–4.5	5.1	4.2	4.2–4.5	3.8	5.6	1.4	**0.0**

**Figure 1.**
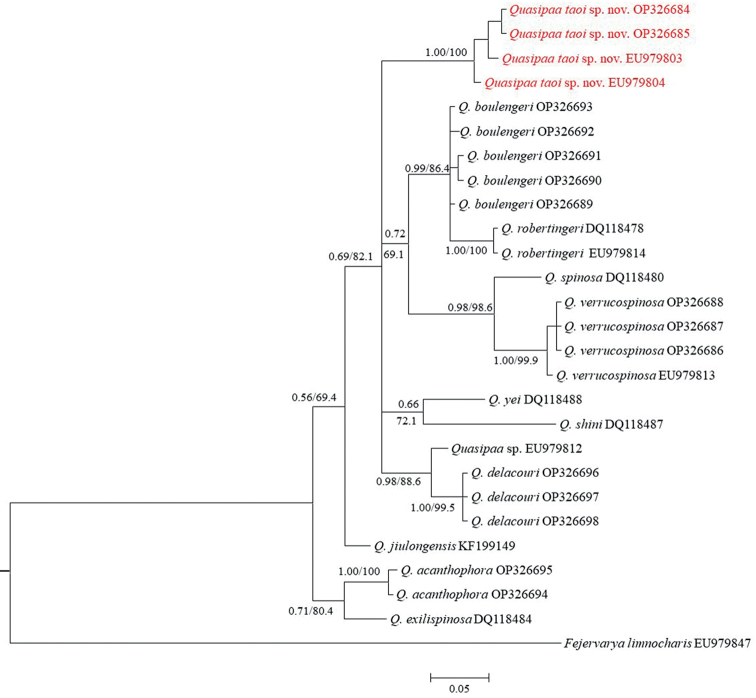
Bayesian phylogram based on a partial 16S mitochondrial fragment. Numbers above and below branches are MP/ML bootstrap values and Bayesian posterior probabilities (>50%), respectively. Hyphen denotes < 50% value. Bold text highlights new samples collected within this study.

The ML and BI analyses produced topologies with –ln L = 1672.0337 and 1729.0216, respectively, with a gamma shape parameter (G: 0.1363 in ML and 0.1767 in BI). Phylogenetic analyses employing ML and BI methods were nearly identical, with most well-supported nodes on the ML tree also well-supported on the BI tree, and only the BI tree is presented in Fig. 1. In both analyses, the newly collected *Quasipaa* specimens from Kon Tum Province were recovered as a separate branch from the *Q.boulengeri* group (*Q.boulengeri*, *Q.robertingeri*, and *Q.verrucospinosa*), the *Q.shini* group (*Q.shini* and *Q.yei*), and the *Q.delacouri* group (*Q.delacouri* and *Quasipaa* sp.).

Our phylogenetic results were in general agreement with those supported by analyses in Che et al. (2009). Although, unlike the topology supported by Zhang et al. (2018), the clade containing the *Q.acanthophora* (haplotypes from Lang Son Province, Vietnam) was recovered as a sister clade to *Q.exilispinosa* with rather strong nodal support from both analyses (0.71/80.4); the clade containing the *Q.delacouri* (haplotypes from Tuyen Quang Province, Vietnam) was recovered as a sister clade to *Quasipaa* sp. from Laos with strong nodal support from both analyses (0.98/98.6) (Fig. 1).

In the following, based on the distinct molecular divergence in concert with diagnostic morphological differences compared to congeners, we describe the *Quasipaa* population from Ngoc Linh based on our integrative taxonomic analysis, as new species to science.

### ﻿Taxonomic account

#### ﻿ Quasipaa taoi sp. nov.

Taxon classificationAnimaliaAnuraDicroglossidae

5F877B8D-C6A8-5A1F-B76C-B46C08BADE22

https://zoobank.org/EEE47B08-108A-49F0-B89E-3512EF353BB1

Figs 2 , 3 , 4

##### Holotype.

IEBR A.4997, adult male, collected by T. Q. Phan and T. D. Tran on 6 March 2019 (15°05'23.3"N, 107°51'17.5"E, at an elevation of 1,560 m asl.) in the evergreen forest of Ngoc Linh Natural Reserve, Xop Commune, Dak Glei District, Kon Tum Province, Vietnam.

##### Paratypes.

IEBR A.4998, adult male; IEBR A.4999, adult male; IEBR A.5000, adult female; IEBR A.5037, adult female; IEBR A.5038, adult female, the same data as the holotype.

##### Diagnosis.

Both morphological characters (body very stout, skin rough with dermal ridges and tubercles, forelimbs of males strongly enlarged, with inner side of arms or fingers or chest and belly with black spines (see Fei et al. 2009) and molecular data revealed the new species to be nested within *Quasipaa*. *Quasipaataoi* sp. nov. is distinguishable from its congeners by a combination of the following morphological characters: (1) SVL 79.6–84.3 mm in males, 64.6–69.9 mm in females; (2) head broader than long (HL/HW 0.90 in males, 0.92 in females); (3) vomerine teeth present; (4) external vocal sacs absent; (5) tympanum slightly visible; (6) dorsum with lines of thick ridges and small round tubercles; (7) flanks covered by oval and round tubercles; (8) supratympanic fold present; (9) dorsolateral fold absent; (10) ventrolateral sides, ventral surface of arms, and all fingers with spines in males; (11) the absence of spines on chest and belly in males; (12) toes fully webbed to distal end of terminal phalanx; (13) in life, dorsum dark brown, chest and belly immaculate white.

**Figure 2.**
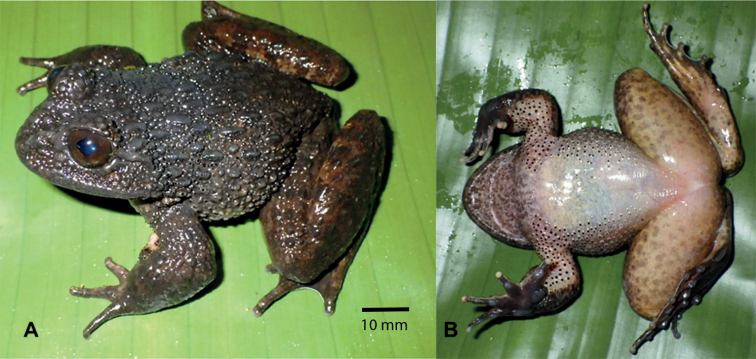
*Quasipaataoi* sp. nov., holotype (IEBR A.4997, male) in life **A** dorsolateral view **B** ventral view.

**Figure 3.**
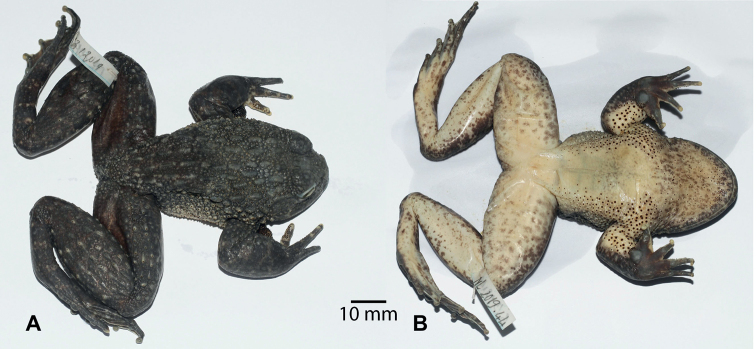
*Quasipaataoi* sp. nov., holotype (IEBR A.4997, male) in preservative **A** dorsolateral view **B** ventral view.

**Figure 4.**
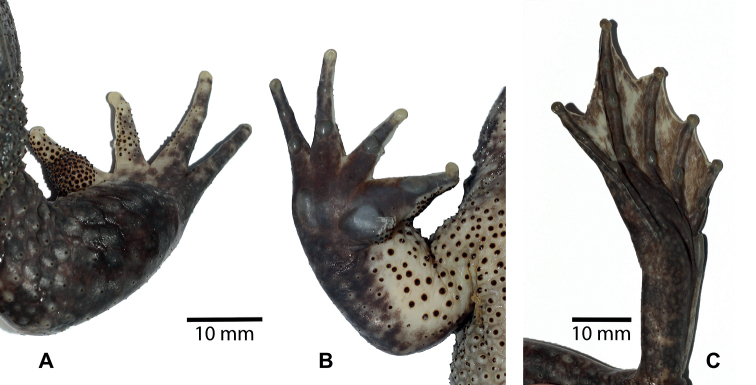
*Quasipaataoi* sp. nov., holotype (IEBR A.4997, male) **A** upper right hand **B** lower right hand **C** lower right foot.

##### Description of holotype.

A large frog (SVL 84.3 mm); habitus robust with enlarged head (HL/SVL 0.40, HW/SVL 0.43); head broader than long (HL 33.5 mm, HW 36.3 mm); snout round anteriorly in dorsal view, projecting beyond lower jaw; nostril lateral, closer to eye than to the tip of snout (NS 7.6 mm, EN 5.5 mm); canthus rostralis indistinct; loreal region oblique and slightly concave; rostral length greater than eye diameter (RL 13.1 mm, ED 9.7 mm); internarial distance wider than interorbital distance and upper eyelid width (IND 8.6 mm, IOD 6.2 mm, UEW 7.7 mm); tympanum slightly visible (TYD 4.1 mm) smaller than the distance from tympanum to eye (TYE 4.9 mm); vomerine teeth in two oblique ridges; tongue cordiform, notched posteriorly; external vocal sac absent.

Forelimbs: arms short; upper arm length (UAL) 17.1 mm, forearm length (FAL) 41.5 mm; relative finger lengths: II<I<IV<III; fingers free of webbing; dermal ridge on sides of fingers present on fingers I, II, III; tips of fingers swollen, not expanded; subarticular tubercles prominent, round, formula 1, 1, 2, 2; inner metatarsal tubercle round; outer metatarsal tubercle elongate; finger I with nuptial pad.

Hindlimbs: tibia length longer than thigh length (FeL 44.2 mm, TbL 49.7 mm), approximately 3.4 times longer than wide (TbW 14.5 mm); tips of toes swollen, slightly round; relative length of toes: I<II<V<III<IV; toes fully webbed to distal end of terminal phalanx; dermal ridge present on outer sides of toes I and V; subarticular tubercles prominent, elongate, formula 1, 1, 2, 3, 2; inner metatarsal tubercle elongate; outer metatarsal tubercle absent; tibio-tarsal articulation reaching to tip of snout.

Skin texture in life: dorsal surface of head with oval and round tubercles, dorsum with six lines of thick ridges intermixed with small round tubercles; flanks covered by oval and round tubercles; supratympanic fold distinct, extending from eye to angle of jaw; dorsolateral fold absent; dorsal surface of forelimbs and hindlimbs with small tubercles; belly and ventral surface of thighs smooth.

Nuptial spines: body of males with spines except for chest, belly, and ventral surface of hindlimbs; dense spines on dorsum, flanks, ventral surface of forelimbs, ventrolateral sides, and fingers I, II; spines present on throat, dorsal surface of fore- and hindlimbs, and fingers III, IV, small and scattered.

Coloration in life: iris dark copper; dorsum and upper part of flanks dark brown; lower part of flanks whitish brown with white tubercles and black spines on top; supratympanic fold dark brown; dorsal surface of limbs yellowish brown with dark crossbars; ventral surface of limbs light yellow with brown markings; throat white with brown markings; chest and belly immaculate white; toe webbing dark brown.

Coloration in preservative: coloration in preservative is the same in life but somewhat faded.

##### Sexual dimorphism.

Measurements and morphological characters of the type series are provided in Table 3. Males are larger than females (SVL 82.7 ± 2.69 mm, *n* = 3 males vs 67.6 ± 2.7 mm, *n* = 3 females). The male specimens have a nuptial pad on finger I and dark spines on flanks, ventral surface of forelimbs, ventrolateral sides, and all fingers. The females contained yellowish-cream eggs of varying sizes.

**Table 3.** Measurements (in mm) and proportions of the type series of *Quasipaaboulengeri* and *Quasipaataoi* sp. nov. (H = holotype, P = paratype, SD = standard deviation; for other abbreviations see Material and methods).

***Quasipaataoi* sp. nov.**										
**Specimen ID**	**IEBR A.4997**	**IEBR A.4999**	**IEBR A.4998**	**Min–Max (*n* = 3)**	**Mean**±**SD (*n* = 3)**	**IEBR A.5000**	**IEBR A.5038**	**IEBR A.5037**	**Min–Max (*n* = 3)**	**Mean**±**SD (*n* = 3)**
Sex	♂	♂	♂			♀	♀	♀		
Type status	H	P	P			P	P	P		
SVL	84.3	79.6	84.2	79.6–84.3	82.7±2.69	68.2	69.9	64.6	64.6–69.9	67.6±2.7
HL	33.5	31.3	32.2	31.3–33.5	32.33±1.11	27.1	28.4	24.9	24.9–28.4	26.8±1.7
HW	36.3	34.9	36.7	34.9–36.7	35.97±0.95	28.3	31.2	27.8	27.8–31.2	29.1±1.8
MN	27.5	26.1	25.9	25.9–27.5	26.5±0.87	22.5	25.0	21.3	21.3–25	22.9±1.9
MFE	22.9	21.9	22.2	21.9–22.9	22.33±0.51	18.8	20.1	17.6	17.6–20.1	18.8±1.3
MBE	13.8	13.7	12.3	12.3–13.8	13.27±0.84	12.1	12.0	9.9	9.9–12.1	11.3±1.2
RL	13.1	12.8	13.4	12.8–13.4	13.1±0.30	11.1	11.5	9.7	9.7–11.5	10.8±0.9
ED	9.7	9.6	10.9	9.6–10.9	10.07±0.72	8.5	7.9	7.7	7.7–8.5	8±0.4
UEW	7.7	7.1	7.6	7.1–7.7	7.47±0.32	6.2	7.4	6.0	6–7.4	6.5±0.8
IND	8.6	7.8	8.5	7.8–8.6	8.3±0.44	7.1	6.2	5.1	5.1–7.1	6.1±1
IOD	6.2	6.1	5.8	5.8–6.2	6.03±0.21	4.8	4.9	4.8	4.8–4.9	4.8±0.1
DAE	12.2	13.2	13.6	12.2–13.6	13.0±0.72	11.3	11.1	9.0	9–11.3	10.5±1.3
DPE	23.7	22.1	23.5	22.1–23.7	23.1±0.87	18.5	20.4	17.8	17.8–20.4	18.9±1.3
NS	7.6	7.5	7.9	7.5–7.9	7.67±0.21	6.5	7.2	6.5	6.5–7.2	6.7±0.4
EN	5.5	5.3	5.4	5.3–5.5	5.4±0.10	4.6	4.9	4.1	4.1–4.9	4.5±0.4
TD	4.1	4.4	4.8	4.1–4.8	4.43±0.35	3.4	4.6	3.8	3.4–4.6	3.9±0.6
TYE	4.9	5	4.9	4.9–5.0	4.93±0.06	4.7	5.0	4.3	4.3–5	4.7±0.4
UAL	17.1	17.6	19.2	17.1–19.2	17.97±1.10	13.6	11.7	11.1	11.1–13.6	12.1±1.3
FAL	41.5	39.9	41.2	39.9–41.5	40.87±0.85	33.2	33.5	29.6	29.6–33.5	32.1±2.1
FeL	44.2	45.3	44.6	44.2–45.3	44.7±0.56	33.8	36.5	33.6	33.6–36.5	34.6±1.6
TbL	49.7	48.9	48.3	48.3–49.7	48.97±0.7	40.1	41.0	37.7	37.7–41	39.6±1.7
TbW	14.5	15.8	15.5	14.5–15.8	15.27±0.68	10.1	12.4	11.4	10.1–12.4	11.3±1.1
FoL	65.6	63.3	64.9	63.3–65.6	64.6±1.18	55.7	54.6	50.1	50.1–55.7	53.5±3
IMT	6.3	5.9	6.1	5.9–6.3	6.1±0.20	4.8	4.6	4.6	4.6–4.8	4.7±0.1
HL/SVL	0.4	0.39	0.38	0.38–0.40	0.39±0.01	0.39	0.39	0.41	0.39–0.41	0.4±0.01
HW/SVL	0.43	0.44	0.44	0.43–0.44	0.43±0.00	0.43	0.41	0.45	0.41–0.45	0.43±0.02
RL/SVL	0.16	0.16	0.16	0.16–0.16	0.16±0.0	0.15	0.15	0.16	0.15–0.16	0.16±0.01
HL/HW	0.92	0.9	0.88	0.88–0.92	0.9±0.02	0.90	0.90	0.96	0.9–0.96	0.92±0.03
ED/RL	0.74	0.75	0.81	0.74–0.81	0.77±0.04	0.79	0.69	0.79	0.69–0.79	0.75±0.06
TYE/TD	1.2	1.14	1.02	1.02–1.20	1.11±0.16	1.13	1.09	1.38	1.09–1.38	1.2±0.16
ED/TD	0.42	0.46	0.44	0.42–0.46	0.44±0.02	0.49	0.4	0.58	0.4–0.58	0.49±0.01
TbL/SVL	0.59	0.61	0.57	0.57–0.61	0.59±0.02	0.58	0.58	0.59	0.58–0.59	0.59±0
TbL/TbW	3.43	3.09	3.12	3.09–3.43	3.21±0.19	3.30	3.30	3.97	3.3–3.97	3.53±0.38
***Q.boulengeri***										
**Specimen ID**	**IEBR A.5007**	**IEBR A.5008**	**IEBR A.5009**	**IEBR A.50109**	**IEBR A.5011**	**IEBR A.5012**	**Min–Max (*n* = 6)**		**Mean**±**SD (*n* = 6)**	
Sex	♂	♂	♂	♂	♂	♂				
Type status										
SVL	100.7	96.8	99.2	101.7	87.8	92.6	87.8–101.7		96.5±5.3	
HL	37.3	37.5	39.8	39.2	35.8	36.3	35.8–39.8		37.6±1.6	
HW	42.0	43.1	45.3	44.8	40.1	40.9	40.1–45.3		42.7±2.1	
MN	31.6	31.7	34.5	34.4	30.2	31.0	30.2–34.5		32.2±1.8	
MFE	26.1	25.9	27.9	28.8	24.7	25.8	24.7–28.8		26.5±1.5	
MBE	17.4	16.4	17.7	19.5	15.5	15.6	15.5–19.5		17±1.5	
RL	14.9	14.3	15.7	14.6	13.9	14.7	13.9–15.7		14.7±0.6	
ED	10.2	10.0	12.4	10.7	10.2	10.4	10–12.4		10.6±0.9	
UEW	8.3	7.4	8.0	7.9	7.3	6.5	6.5–8.3		7.6±0.7	
IND	8.3	7.9	9.3	8.2	8.3	8.0	7.9–9.3		8.3±0.5	
IOD	6.6	5.9	8.4	8.2	5.5	7.5	5.5–8.4		7±1.2	
DAE	14.7	14.3	15.8	15.6	13.0	13.5	13–15.8		14.5±1.1	
DPE	26.1	25.4	29.6	28.4	24.3	26.0	24.3–29.6		26.6±2	
NS	9.1	7.0	7.7	7.4	7.7	7.8	7–9.1		7.8±0.7	
***Q.boulengeri***										
**Specimen ID**	**IEBR A.5007**	**IEBR A.5008**	**IEBR A.5009**	**IEBR A.50109**	**IEBR A.5011**	**IEBR A.5012**	**Min–Max (*n* = 6)**		**Mean**±**SD (*n* = 6)**	
EN	6.3	6.7	8.2	7.3	5.8	6.5	5.8–8.2		6.8±0.8	
TD	6.5	6.0	6.7	6.3	6.1	5.7	5.7–6.7		6.2±0.4	
TYE	5.6	5.6	7.1	6.2	5.0	6.2	5–7.1		5.9±0.7	
UAL	20.0	19.3	21.0	20.0	15.4	15.7	15.4–21		18.6±2.4	
FAL	46.0	49.6	51.9	52.8	46.5	45.7	45.7–52.8		48.7±3.1	
FeL	54.1	50.3	57.0	52.3	48.2	48.7	48.2–57		51.8±3.4	
TbL	53.9	52.6	56.3	57.4	49.7	52.2	49.7–57.4		53.7±2.8	
TbW	16.8	17.7	17.7	19.0	16.5	17.3	16.5–19		17.5±0.9	
FoL	72.5	72.3	77.3	78.6	67.8	68.7	67.8–78.6		72.9±4.4	
IMT	8.1	8.1	7.4	8.8	7.7	8.0	7.4–8.8		8±0.5	
HL/SVL	0.37	0.39	0.40	0.39	0.41	0.39	0.37–0.41		0.4±0	
HW/SVL	0.42	0.45	0.46	0.44	0.46	0.44	0.42–0.46		0.4±0	
RL/SVL	0.15	0.15	0.16	0.14	0.16	0.16	0.14–0.16		0.2±0	
HL/HW	0.89	0.87	0.88	0.88	0.89	0.89	0.87–0.89		0.9±0	
ED/RL	0.69	0.70	0.79	0.73	0.73	0.71	0.69–0.79		0.7±0	
TYE/TD	0.86	0.93	1.05	0.98	0.82	1.09	0.82–1.09		1±0.1	
ED/TD	0.64	0.60	0.55	0.59	0.60	0.55	0.55–0.64		0.59±0	
TbL/SVL	0.54	0.54	0.57	0.56	0.57	0.56	0.54–0.57		0.6±0	
TbL/TbW	3.21	2.97	3.18	3.02	3.02	3.01	2.97–3.21		3.1±0.1	
***Q.boulengeri***										
**Specimen ID**	**IEBR A.5013**	**IEBR A.5014**	**IEBR A.5039**	**IEBR A.5040**	**IEBR A.5041**	**Min–Max (*n* = 5)**		**Mean**±**SD (*n* = 5)**		
Sex	♀	♀	♀	♀	♀					
Type status										
SVL	105.5	95.5	90.8	82.5	91.4	82.5–105.5		93.1±8.4		
HL	39.9	36.4	34.1	30.3	34.9	30.3–39.9		35.1±3.5		
HW	44.2	39.5	39.4	39.4	41.0	39.4–44.2		40.7±2.1		
MN	32.9	30.0	28.1	25.5	30.8	25.5–32.9		29.5±2.8		
MFE	26.7	24.4	22.1	19.8	25.0	19.8–26.7		23.6±2.7		
MBE	17.6	13.7	13.7	11.9	16.1	11.9–17.6		14.6±2.2		
RL	15.7	13.7	13.0	11.4	12.6	11.4–15.7		13.3±1.6		
ED	10.8	11.4	9.4	9.1	10.6	9.1–11.4		10.3±1		
UEW	7.7	7.3	7.6	6.7	7.9	6.7–7.9		7.4±0.5		
IND	9.4	7.8	8.0	7.5	9.0	7.5–9.4		8.3±0.8		
IOD	8.5	6.0	5.9	6.1	5.4	5.4–8.5		6.4±1.2		
DAE	15.8	13.5	12.8	13.0	12.2	12.2–15.8		13.4±1.4		
DPE	28.6	24.8	13.8	21.9	9.0	9–28.6		19.6±8		
NS	9.3	7.3	7.6	6.9	8.2	6.9–9.3		7.9±0.9		
EN	6.1	6.6	5.6	5.3	5.8	5.3–6.6		5.9±0.5		
TD	6.7	5.4	5.1	5.2	6.1	5.1–6.7		5.7±0.7		
TYE	6.6	4.9	5.0	3.8	5.7	3.8–6.6		5.2±1		
UAL	16.4	16.6	15.3	11.8	15.1	11.8–16.6		15±1.9		
FAL	47.7	43.0	40.6	36.7	43.7	36.7–47.7		42.3±4.1		
FeL	52.0	47.7	46.6	41.2	46.6	41.2–52		46.8±3.8		
TbL	54.0	50.5	47.8	44.2	49.8	44.2–54		49.3±3.6		
TbW	18.7	18.1	17.8	14.9	16.5	14.9–18.7		17.2±1.5		
FoL	74.4	67.6	65.4	60.0	69.2	60–74.4		67.3±5.3		
IMT	8.1	6.8	16.1	5.5	7.8	5.5–16.1		8.9±4.2		
HL/SVL	0.38	0.38	0.38	0.37	0.38	0.4–0.4		0.4±0		
HW/SVL	0.42	0.41	0.43	0.48	0.45	0.4–0.5		0.4±0		
RL/SVL	0.15	0.14	0.14	0.14	0.14	0.1–0.1		0.1±0		
HL/HW	0.90	0.92	0.87	0.77	0.85	0.8–0.9		0.9±0.1		
ED/RL	0.69	0.83	0.72	0.80	0.84	0.7–0.8		0.8±0.1		
TYE/TD	0.99	0.92	0.98	0.74	0.94	0.7–1		0.9±0.1		
ED/TD	0.62	0.47	0.54	0.57	0.58	0.47–0.62		0.56±0		
TbL/SVL	0.51	0.53	0.53	0.54	0.54	0.5–0.5		0.5±0		
TbL/TbW	2.88	2.79	2.69	2.96	3.02	2.7–3		2.9±0.1		

##### Etymology.

The new species is named in honor of our colleague and friend, Assoc. Prof. Dr. Tao Thien Nguyen from the Institute of Genome Research, Vietnam Academy of Science and Technology, in recognition of his numerous scientific contributions towards a better understanding of the amphibians of Vietnam. We recommend “Tao’s Spiny Frog” as the common English name of the new species and the common name in Vietnamese as “Ếch gai sần tạo”.

##### Ecological notes.

**Figure 5.**
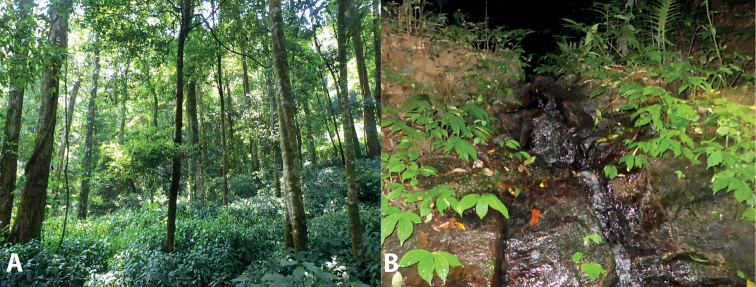
Habitat of *Quasipaataoi* sp. nov. in Ngoc Linh Nature Reserve, Kon Tum Province, Viet Nam **A** evergreen forest **B** microhabitat.

Specimens were found between 19:00 and 23:00 in the headwaters of rocky streams (Fig. 5B). They were found in the water or on the ground of stream banks at an elevation of above 1,500 m a.s.l. The surrounding habitat was secondary forest of large, medium-sized, and small hardwoods mixed with shrubs and vines (Fig. 5A). Air temperatures at the sites ranged from 18.5–22.5 °C and relative humidity was 68–85%. Male advertisement calls and tadpoles of the species have not been recorded during our field surveys. Other amphibian species found at the sites included *Leptobrachella* sp., *Limnonecteskiziriani* Pham, Le, Ngo, Ziegler & Nguyen, 2018, *L.poilani* (Bourret, 1942), *Amolopsspinapectoralis* Inger, Orlov & Darevsky, 1999, *Odorranakhalam* (Stuart, Orlov & Chan-ard, 2005), *O.morafkai* (Bain, Lathrop, Murphy, Orlov & Ho, 2003), Kurixaluscf.banaensis (Bourret, 1939), and *Rhacophorusannamensis* (Smith, 1924).

##### Distribution.

**Figure 6.**
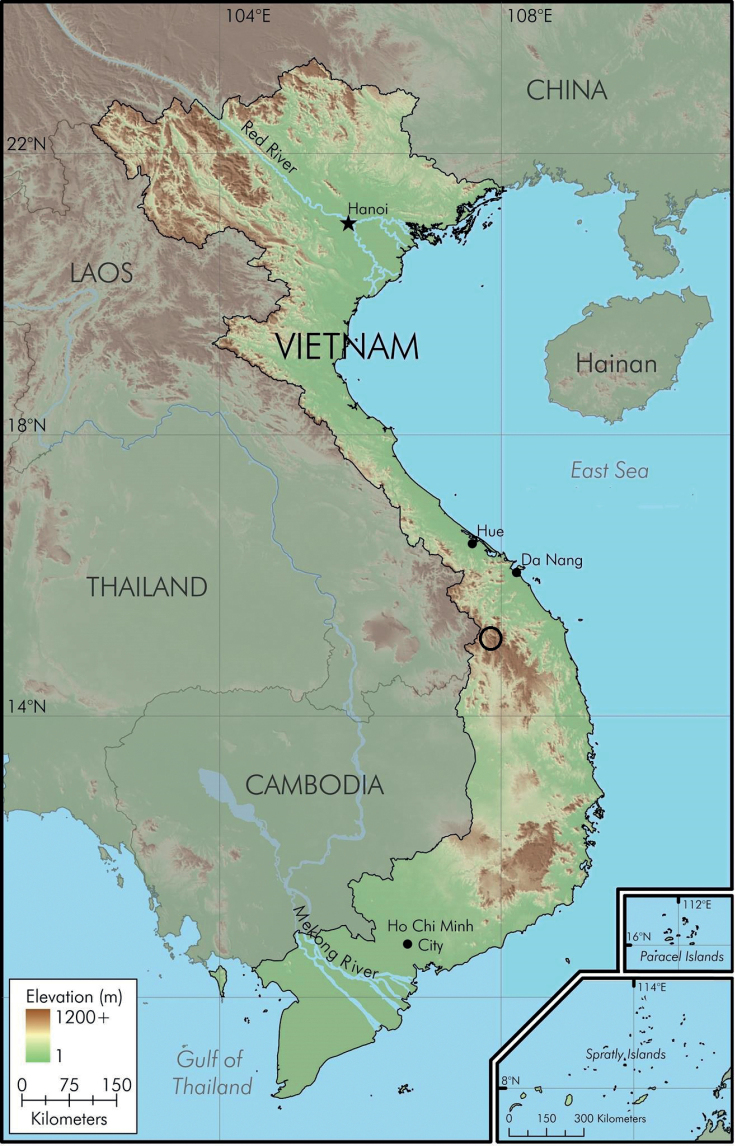
Map showing the type locality (circle) of *Quasipaataoi* sp. nov. in Kon Tum Province, Vietnam.

*Quasipaataoi* sp. nov. is currently known from Ngoc Linh Mountain of the Central Highlands in Vietnam (Fig. 6). Data obtained from GenBank show that this species was also recorded from Xekong Province, Lao PDR (Yan et al. 2021; see Discussion below).

##### Comparisons.

**Figure 7.**
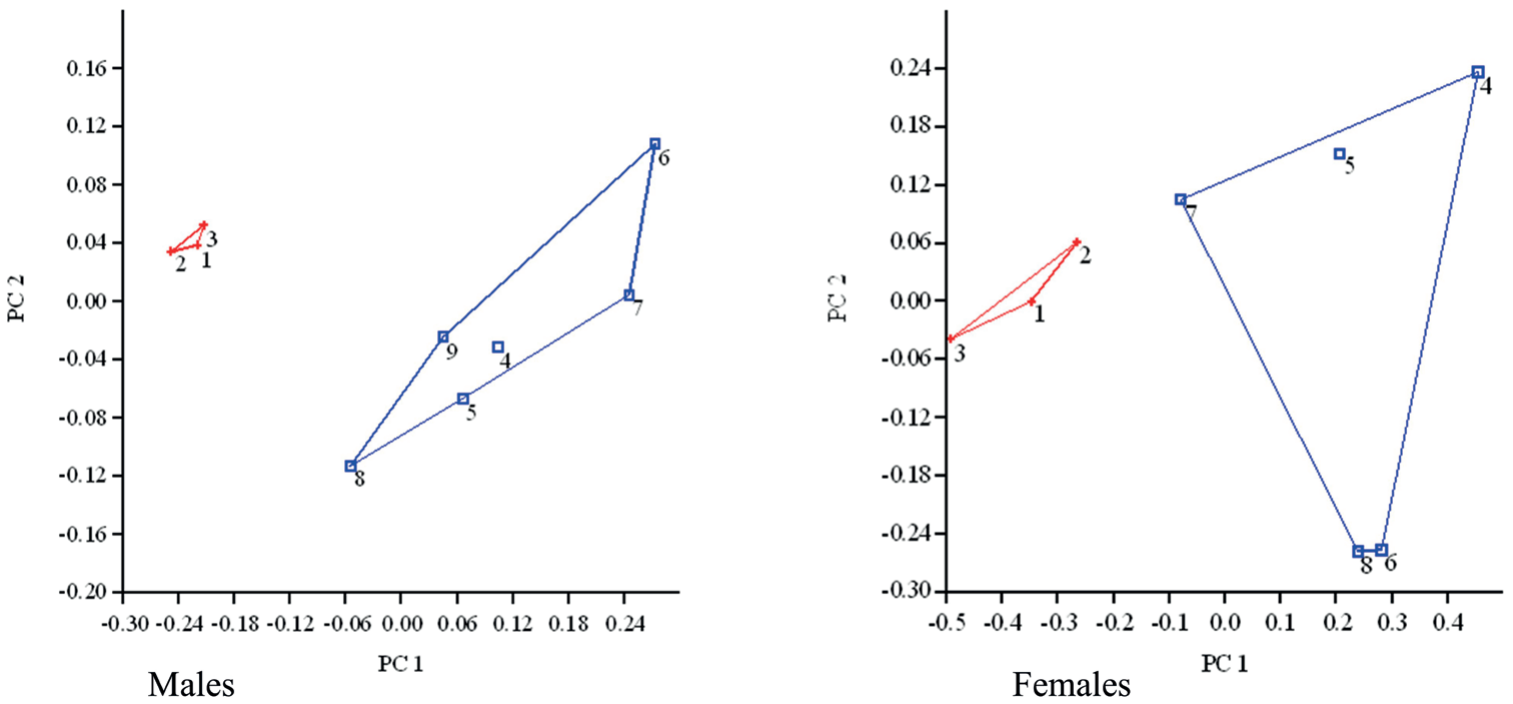
Plots of the first principal component (PC1) versus the second (PC2) for the males and the females of *Quasipaataoi* sp. nov. (red +) and *Q.boulengeri* (blue □).

We compared the new species with its congeners. *Quasipaataoi* sp. nov. differs from *Q.boulengeri* by having a smaller size, SVL 79.6–84.3 mm, *n* = 3 in males, 64.6–69.9 mm, *n* = 3 in females (vs 87.8–101.7 mm, *n* = 6 in males, 82.5–105.5 mm, *n* = 5 in females), dorsum with thick ridges and round tubercles (vs elongate ridges), males with nuptial spines on all fingers (vs absent on finger IV); males with nuptial spines on throat and ventral surface of arms (vs absent), and the absence of nuptial spines on chest and belly in males (vs present). In the PCA analysis, the first two principal component axes could separate *Quasipaataoi* sp. nov. from *Q.boulengeri* by 24 characters (Fig. 7), mainly based on limb and head measurements, namely: SVL, HW, HL, MN, MFE, MBE, RL, ED, UEW, IND, IOD, DAE, DPE, NS, EN, TD, TYE, UAL, FAL, FeL, TbL, TbW, FoL, and IMT (Tables 3, 4). In males, the PCA extracted three principal component axes with eigenvalues greater than 0.002 and, of these, the first two component axes accounted for 85.50% of the variation (Table 4). Species with a larger and positive score on PC1 reflected shorter SVL including all traits. The PC2 with positive scores were associated with species having greater measurements of RL, ED, UEW, IND, IOD, DAE, DPE, NS, EN, TYE, UAL, FeL, TbL, and FoL, while negative scores with species having smaller measurements of SVL, HW, HL, MN, MFE, MBE, TD, FAL, TbW, and IMT (Table 4). In females, the PCA extracted three principal component axes with eigenvalues greater than 0.01 and of these, the first two component axes accounted for 85.98% of the variation (Table 4). Species with a higher and positive score on PC1 reflected having shorter measurements of SVL, HW, HL, MN, MFE, MBE, RL, ED, UEW, IND, IOD, DAE, NS, EN, TD, TYE, UAL, FAL, FeL, TbL, TbW, FoL, and IMT, while a negative score with species having smaller DPE. The PC2 with positive scores were associated with species having greater measurements of SVL, HW, HL, MN, MFE, MBE, RL, ED, IOD, DAE, DPE, NS, EN, TD, TYE, UAL, FAL, FeL, TbL, TbW, and FoL, while a negative score with species having smaller measurements of UEW, IND, and IMT (Table 4). *Quasipaataoi* sp. nov. differs from *Q.acanthophora* by having the dorsum with thick ridges and round tubercles (vs small tubercles), males with nuptial spines on ventrolateral sides and ventral surface of arms (vs absent), males with nuptial spines on all fingers (vs absent on finger IV), and the absence of spines on chest of males (vs present). *Quasipaataoi* sp. nov. differs from *Q.courtoisi* by having a smaller size in males, SVL 79.6–84.3 mm, *n* = 3 (vs 126 mm, *n* = 1); males with nuptial spines on throat and ventral surface of arms (vs absent); and the absence of nuptial spines on chest in males (vs present). *Quasipaataoi* sp. nov. differs from *Q.delacouri* by having a smaller size, SVL 79.6–84.3 mm, *n* = 3, in males and 64.6–69.9 mm, *n* = 3, in females (vs 92.9–115.5 mm, *n* = 4, in males and 94.5–117.5 mm, *n* = 3, in females); a greater ratio of TD/ED, 0.44 ± 0.02, *n* = 3, in males and 0.49 ± 0.01, *n* = 3, in females (vs 0.26 in males and 0.24 in females); dorsum with thick ridges and round tubercles (vs smooth); males with nuptial pad on finger I (vs absent in males); and males with nuptial spines (vs absent). The new species differs from *Q.exilispinosa* by having a larger size in males (SVL 79.6–84.3 mm, *n* = 3, in males and 64.6–69.9 mm, *n* = 3, in females (vs 61.2 mm, *n* = 20, in males and 57.1 mm, *n* = 20, in females); dorsum with thick ridges and round tubercles (vs small tubercles); males with nuptial spines on ventrolateral sides and ventral surface of arms (vs absent); males with nuptial spines on all fingers (vs absent on finger IV); and absence of spines on chest in males (vs present). *Quasipaataoi* sp. nov. differs from *Q.fasciculispina* by having a smaller size, SVL 79.6–84.3 mm, *n* = 3 in males and 64.6–69.9 mm, *n* = 3 in females (vs 106 mm, *n* = 1 in males and 104 mm, *n* = 1 in females); a smaller ratio of TYE/TD (1.11 ± 0.16, *n* = 3, in males and 1.2 ± 0.16, *n* = 3, in females (vs 2.0 in male and 1.75 in female); the absence of nuptial spines on chest in males (vs circular whitish tubercles each bearing 5–10 strong black spines). *Quasipaataoi* sp. nov. differs from *Q.jiulongensis* by having dorsum with thick ridges and round tubercles (vs small tubercles), males with nuptial spines on ventrolateral sides and ventral surface of arms of males (vs absent), males with nuptial spines on all fingers (vs absent on fingers III and IV); the absence of light-colored longitudinal stripes on upper jaw edge (vs present); and the absence of 4 or 5 yellow dorsal dots arranged in longitudinal rows (vs present). *Quasipaataoi* sp. nov. differs from *Q.robertingeri* by having dorsum with thick ridges and round tubercles (vs elongate ridges), males with nuptial spines on all fingers (vs absent on finger IV); males with nuptial spines on throat and ventral surface of arms (vs absent), and the absence of nuptial spines on chest and belly of males (vs present). *Quasipaataoi* sp. nov. differs from *Q.shini* by its smaller size, SVL 79.6–84.3 mm, *n* = 3, in males and 64.6–69.9 mm, *n* = 3, in females (vs 98.6 mm, *n* = 9, in males and 94.9 mm, *n* = 10, in females); dorsum with thick ridges and round tubercles (vs elongate ridges), males with nuptial spines on all fingers (vs absent on finger IV); males with nuptial spines on throat and ventral surface of arms (vs absent), and the absence of nuptial spines on chest and belly of males (vs present). *Quasipaataoi* sp. nov. differs from *Q.spinosa* by its smaller size, SVL 79.6–84.3 mm, *n* = 3, in males and 64.6–69.9 mm, *n* = 3, in females (vs 106.0–142.0 mm, *n* = 20, in males and 115.0–152.5 mm, *n* = 10, in females); dorsum with short, thick ridges and round tubercles (vs small tubercles); the absence of light colored longitudinal stripes on upper jaw edge (vs present); and the absence of nuptial spines on chest of males (vs small and dense spines on chest of males). *Quasipaataoi* sp. nov. differs from *Q.verrucospinosa* by its smaller size (SVL 79.6–84.3 mm, *n* = 3, in males and 64.6–69.9 mm, *n* = 3, in females (vs 90.0–117.0, *n* = 8, in males, 83.2–113.9 mm, *n* = 9, in females); males with nuptial spines on all fingers (vs absent on fingers III and IV); males with nuptial spines on ventral surface of arms (vs absent), and the absence of nuptial spines on chest and belly in males (vs present). *Quasipaataoi* sp. nov. differs from *Q.yei* by its larger size in males (SVL 79.6–84.3 mm, *n* = 3, in males and 64.6–69.9 mm, *n* = 3, in females (vs 49.7–64.0 mm, *n* = 25); males with nuptial spines on ventrolateral sides and ventral surface of arms (vs absent); and males with nuptial spines on all fingers (vs absent); absence of nuptial spines around and inside vent (vs present).

**Table 4.** Variable loadings for principal components with eigenvalue greater than 0.01, from morphometric characters corrected by SVL. All measurements were given in millimeter (mm).

	Male			Female		
	Axis 1	Axis 2	Axis 3	Axis 1	Axis 2	Axis 3
Eigenvalue	0.039	0.004	0.003	0.118	0.032	0.012
% variance	76.63	8.86	5.75	67.42	18.57	7.00
SVL	0.189	−0.066	0.117	0.223	0.055	0.012
HW	0.205	−0.077	0.039	0.216	0.033	0.041
HL	0.181	−0.068	0.005	0.202	0.056	0.047
MN	0.234	−0.083	−0.055	0.189	0.033	0.123
MFE	0.212	−0.056	−0.058	0.178	0.038	0.157
MBE	0.300	−0.154	−0.016	0.212	0.017	0.224
RL	0.139	0.009	0.021	0.173	0.100	0.005
ED	0.106	0.258	0.065	0.164	0.052	0.117
UEW	0.056	0.148	0.460	0.111	−0.035	0.094
IND	0.039	0.216	0.112	0.229	−0.002	0.166
IOD	0.261	0.479	−0.442	0.195	0.207	−0.098
DAE	0.167	0.138	0.169	0.182	0.150	−0.012
DPE	0.198	0.109	−0.036	−0.008	0.825	−0.441
NS	0.008	0.013	0.227	0.134	0.027	0.108
EN	0.310	0.163	−0.108	0.180	0.069	0.059
TD	0.359	−0.322	0.254	0.258	0.063	0.198
TYE	0.258	0.241	-0.279	0.145	0.012	0.215
UAL	0.125	0.373	0.530	0.177	0.000	0.019
FAL	0.213	−0.084	-0.008	0.204	0.035	0.100
FeL	0.185	0.027	0.143	0.211	0.028	0.006
TbL	0.131	0.053	−0.008	0.158	0.034	0.055
TbW	0.161	−0.094	−0.082	0.280	0.023	−0.091
FoL	0.164	0.026	0.061	0.169	0.022	0.076
IMT	0.271	−0.457	−0.078	0.413	−0.466	−0.724

## ﻿Discussion

Mount Ngoc Linh, on the northwestern border of the Kon Tum Massif, is the highest peak in central Vietnam at 2,598 m (Sterling et al. 2006). Ngoc Linh is the type locality of several new species of amphibians, namely *Leptobrachiumngoclinhense* (Orlov, 2005), *Thelodermanebulosum* Rowley, Le, Hoang, Dau & Cao, 2011, *Leptobrachellafirthi* (Rowley, Hoang, Dau, Le & Cao, 2012); *Gracixaluslumarius* Rowley, Le, Dau, Hoang & Cao, 2014, *G.trieng* Rowley, Le, Hoang, Cao & Dau, 2020 (Orlov 2005, Rowley et al. 2011, 2012, 2014, 2020). Most recently, Krzikowski et al. (2022) highlighted the extraordinary endemism rate of amphibians in the Central Highlands of Vietnam and, thus, the special role in amphibian diversification and evolution. A number of amphibian species are currently known only from this region, namely *Leptobrachellacrocea* (Rowley, Hoang, Le, Dau & Cao, 2010), *Leptobrachiumngoclinhense*; *Microhyladarevskii* Poyarkov, Vassilieva, Orlov, Galoyan, Tran, Le, Kretova, & Geissler, 2014, *Gracixaluslumarius*, *G.trieng*, and *Thelodermanebulosum* (Frost 2022; Krzikowski et al. 2022). *Quasipaataoi* represents the thirteenth known species of *Quasipaa* and the sixth known species of this genus from Vietnam (Frost 2022; this study). Further studies, as a result, will likely uncover more cryptic species in this poorly known group of frogs.

The new species has a restricted distribution in central Vietnam and Xekong Province, Lao PDR. A major threat to the new species in the area is habitat loss by agricultural extension for medicinal trees (e.g. *Panaxvietnamensis*), illegal timber logging, and tourism development. In addition, the species *Q.taoi* is collected by local people for food. We suggest assessment of this species as Near Threatened in the IUCN Red List of Threatened Species because the continued survival of this species is largely dependent on the protection and rigorous management provided by local authorities of the protected areas in both countries.

In this study, we first uploaded to GenBank the 16S gene sequence of *Quasipaaacanthophora* from the type locality (Mau Son Mountain) in Lang Son Province, northern Vietnam. We confirm that *Q.acanthophora* is currently known only from Vietnam and does not correpond to a population of *Q.spinosa* according to Yan et al. (2021). Based on morphological comparisons, we also provided the 16S gene sequences of true *Quasipaadelacouri* from Tuyen Quang Province, near the type locality in Bac Kan Province, Vietnam. This will assist in clarifying the taxonomy of species in the genus *Quasipaa* in the future.

## Supplementary Material

XML Treatment for Quasipaa taoi

## ﻿Appendix 1

Specimens examined

*Quasipaaacanthophora* (*n* = 11): Vietnam: Lang Son Province: Cao Loc District: Mau Son Commune: IEBR A.5030–A.5035; Vietnam: Bac Giang Province: Son Dong District: Tay Yen Tu Nature Reserve: IEBR A.2013.62–A.2013.64, IEBR 3625–3626.

*Quasipaaboulengeri* (*n* = 13): Vietnam: Cao Bang Province: Nguyen Binh District: Phia Oac-Phia Den National Park: IEBR A.5007–A.5014, IEBR A.5039–A.5041; Vietnam: Ha Giang Province: Quan Ba District: IEBR A.5015–A.5016.

*Quasipaadelacouri* (*n* = 4): Vietnam: Tuyen Quang Province: Cham Chu Nature Reserve: IEBR A.5017; Vietnam: Ha Giang Province: Bac Me Nature Reserve: IEBR A.5018; Vietnam: Phu Tho Province: IEBR A.5019–A.5020.

*Quasipaaverrucospinosa* (*n* = 9): Vietnam: Vinh Phuc Province: Tam Dao National Park: IEBR A.5021; Vietnam: Ha Giang Province: Bac Me Nature Reserve: IEBR A.5022–A.5024; Vietnam: Tuyen Quang Province: Cham Chu Nature Reserve: IEBR A.5025–A.5027; Vietnam: Tuyen Quang Province: Na Hang District: IEBR A.5028–A.5029.
